# Selection of suitable reference genes for normalization of quantitative RT-PCR (RT-qPCR) expression data across twelve tissues of riverine buffaloes (*Bubalus bubalis*)

**DOI:** 10.1371/journal.pone.0191558

**Published:** 2018-03-06

**Authors:** Ramneek Kaur, Monika Sodhi, Ankita Sharma, Vijay Lakshmi Sharma, Preeti Verma, Shelesh Kumar Swami, Parvesh Kumari, Manishi Mukesh

**Affiliations:** 1 Department of Animal Biotechnology, ICAR-National Bureau of Animal Genetic Resources, Karnal, Haryana, India; 2 Department of Zoology, Panjab University, Chandigarh, India; Nazarbayev University, KAZAKHSTAN

## Abstract

Selection of reference genes has become an integral step in any real time quantitative PCR (RT-qPCR) based expression studies. The importance of this study stems from the fact that riverine buffaloes are major dairy species of Indian sub-continent and the information generated here will be of great interest to the investigators engaged in functional genomic studies of this important livestock species. In this study, an effort was made to evaluate a panel of 10 candidate reference genes (glyceraldehyde 3-phosphate dehydrogenase (*GAPDH)*, beta- actin (*ACTB*), ubiquitously expressed transcript (*UXT*), ribosomal protein S15 (*RPS15*), ribosomal protein L-4 (*RPL4*), ribosomal protein S9 (*RPS9*), ribosomal protein S23 (*RPS23*), hydroxymethylbilane synthase (*HMBS*), β2 Microglobulin (*β2M*) and eukaryotic translation elongation factor 1 alpha 1 (*EEF1A1*) across 12 tissues (mammary gland, kidney, spleen, liver, heart, intestine, ovary, lung, muscle, brain, subcutaneous fat and testis) of riverine buffaloes. In addition to overall analysis, tissue wise evaluation of expression stability of individual RG was also performed. Three different algorithms provided in geNorm, NormFinder and BestKeeper softwares were used to evaluate the stability of 10 potential reference genes from different functional classes. The M-value given by geNorm ranged from 0.9797 (*RPS9* and *UXT*) to 1.7362 (*RPS15*). From the most stable to the least stable, genes were ranked as: *UXT/RPS9> RPL4> RPS23> EEF1A1> ACTB> HMBS> GAPDH> B2M> RPS15*. While NormFinder analysis ranked the genes as: *UXT> RPS23> RPL4> RPS9> EEF1A1> HMBS> ACTB> β2M> GAPDH> RPS15*. Based on the crossing point SD value and range of fold change expression, BestKeeper analysis ranked the genes as: *RPS9> RPS23/UXT> RPL4> GAPDH> EEF1A1> ACTB> HMBS> β2M> RPS15*. Overall the study has identified *RPS23*, *RPS9*, *RPL4* and *UXT* genes to be the most stable and appropriate RGs that could be utilized for normalization of transcriptional data in various tissues of buffaloes. This manuscript thus provide useful information on panel of reference genes that could be helpful for researchers conducting functional genomic studies in riverine buffaloes.

## Introduction

The riverine buffalo (*Bubalus bubalis*) is major livestock species of India that plays an important role in agricultural economy due to its dairy, meat and draught potential. It contributes more than half of the total milk production of India [1]. As buffalo is the major livestock species and milk contributor for Indian subcontinent, therefore concerted efforts on genomic and transcriptomic studies to determine gene functions, gene networks and biological pathways for various phenomic traits needs to be strengthened. Understanding expressed genes pattern is critical to provide insights into complex regulatory networks and for identification of genes relevant to new biological processes. Among the array of techniques available, quantitative real time polymerase chain reaction (qPCR) is a well-established and widely used technique to quantify precise mRNA levels of target gene of interest in different biological samples. Due to its several benefits over the conventional methods of measuring RNA, like higher sensitivity, large dynamic range and potential for high throughput, the technique has revolutionized the approach to analyze the gene expression patterns in various fields of biological research [2–4]. However the expression data from qPCR studies needs normalization as this technique is prone to analytical variations. To achieve accurate quantification, it is quite essential to take into account the variations that might occur due to differing amount of starting material, pipetting errors, efficiencies of RNA extraction and reverse transcription. Use of internal control genes (ICGs) or reference genes (RGs) with constant expression level between samples in response to experimental treatment or physiological state, are now considered as effective method for normalization of transcriptional data to account for the experimental variations. This approach has been widely used in different cell types and tissues in various species [5–7]. In past though, trend was to use only single reference gene; mostly *GAPDH*, *ACTB* or *18S rRNA* to normalize the real time PCR data. However, now it is an established notion that for each experimental set-up, proper evaluation of multiple RGs is crucial to avoid variations and use of single reference gene for normalization of qPCR data is inappropriate [8]. Therefore, number of studies have successfully been conducted to identify least regulated and stably expressed RGs [9–11] across tissues of various species such as human, pig, sheep, bovines *etc* [12–18]. To our knowledge, not much information is available on set of suitable reference genes that can be used in lactation or physiological studies in riverine buffaloes (*Bubalus bubalis*) although few studies have been performed in specific cell types/tissues [19–21]. Considering the fact that choice of suitable reference/RGs genes is crucial for accurate expression profiling of target genes, this study presents the information on panel of appropriate RGs across 12 buffalo tissues. Such information will be a useful resource to normalize the qPCR based expression data generated across tissues during various physiological or metabolic studies in riverine buffaloes.

## Materials and methods

### Collection of tissue samples

Twelve tissue samples *viz*; Mammary gland, kidney, spleen, liver, heart, intestine, ovary, lung, muscle, brain, subcutaneous fat and testis from 5 adult riverine buffaloes were collected from Ghazipur abattoir, New Delhi. Immediately after collection, the tissue samples were snap frozen in liquid N_2_ and immediately transferred to laboratory. In total, 60 samples of 12 different tissues were collected for RNA extraction and cDNA synthesis.

### Total RNA extraction and cDNA synthesis

Total RNA was extracted from 60 samples representing 12 tissues (mammary gland, kidney, spleen, liver, heart, intestine, ovary, lung, muscle, brain, subcutaneous fat and testis) from 5 adult riverine buffaloes using ice-cold Trizol reagent (Invitrogen, USA) according to the manufacturer’s protocol. After extraction, RNA was purified using RNeasy Mini kit (Qiagen, Germany) and then subsequently followed by on-column digestion with the RNase-free DNase (Qiagen). The RNA was quantified using Nanodrop ND-1000 spectrophotometer (NanoDrop Technologies). RNA integrity was confirmed by denaturing agarose gel electrophoresis as well as checking the RIN value of representative RNA samples using experion Bioanalyser (BioRad, USA). The RNA samples were stored at -80°C till further use. First strand cDNA was synthesized using 100ng RNA as described in our previous studies [7,19] using the program: 25°C for 5 min, 50°C for 60 min, and 70°C for 15 min. cDNA was then diluted 1:4 (v:v) with DNase/RNase free water. Sufficient cDNA was prepared in a single run to perform the qPCR experiments for all selected genes. The primer details for reference genes are given in Table 1. Primers specificity was confirmed in 20 μL PCR reaction using the similar protocol as described for qPCR except for the final dissociation protocol. 5 μL of the PCR product was run in a 2% agarose gel stained with ethidium bromide. The accuracy of primer pairs was also ensured by the presence of a unique peak during the dissociation step at the end of qPCR. PCR efficiency (E) for each primer pair was calculated on the basis of slope of standard curve using equation: E = 10^(1/-slope)^.

**Table 1:** Gene name, gene symbol, GenBank accession numbers, primer sequences, primer location, annealing temperature (T_a_) and amplicon length for each evaluated RGs.

Gene Name	Gene Symbol	Accession Number	Primers 5'-3' (Forward, Reverse)	Primer Location	T_a_	Amplicon Size (bp)
β- actin	*ACTB*	AY141970	GCGTGGCTACAGCTTCACC TTGATGTCACGGACGATTTC	EXON 4	60°C	56
Glyceraldehyde 3-phosphate dehydrogenase	*GAPDH*	BC102589	TGGAAAGGCCATCACCATCT CCCACTTGATGTTGGCAG	EXON 4–5	60°C	60
Eukaryotic translation elongation factor 1 alpha 1	*EEF1A1*	BC105315	CATCCCAGGCTGACTGTGC TGTAAGCCAAAAGGGCATGC	EXON 3–4	60°C	101
β2 Microglobulin	*B2M*	NM_173893	CTGCTATGTGTATGGGTTCC GGAGTGAACTCAGCGTG	EXON 2	60°C	101
Hydroxymethylbilane synthase	*HMBS*	BC112573.1	CTTTGGAGAGGAATGAAGTGG AATGGTGAAGCCAGGAGGAA	EXON 5–6	60°C	80
Ribosomal protein L4	*RPL4*	NM_001014894	TTGGAAACATGTGTCGTGGG GCAGATGGCGTATCGCTTCT	EXON 3–4	60°C	101
Ribosomal protein S15	*RPS15*	BC108231	GAATGGTGCGCATGAATGTC GACTTTGGAGCACGGCCTAA	EXON 2	60°C	101
Ribosomal protein S23	*RPS23*	BC102049	CCCAATGATGGTTGCTTGAA CGGACTCCAGGAATGTCACC	EXON 3–4	60°C	101
Ribosomal protein S9	*RPS9*	DT860044	CCTCGACCAAGAGCTGAAG CCTCCAGACCTCACGTTTGTTC	EXON 2–3	60°C	54
Ubiquitously expressed transcript	*UXT*	CR452243	TGTGGCCCTTGGATATGGTT GGTTGTCGCTGAGCTCTGTG	EXON 5–6	60°C	101

### Real-time quantitative PCR (qPCR)

The qPCR reactions were performed in a final volume of 10 μL containing 4 μL diluted cDNA combined with 6 μL of master mix composed of 5 μL Maxima SYBR Green/ROX qPCR master mix (2X) (Fermentas Thermo, USA), 0.4 μL each of 10 μM forward and reverse primers, and 0.2 μL DNase/RNase free water. The reactions set up in Step one plus real time PCR instrument (ABI, California) were performed at 2 min at 50°C, 10 min at 95°C, 40 cycles of 15 s at 95°C (denaturation) and 1 min at 60°C (annealing+extension). The standard curves were made with 5 point relative standard curve of five-fold serial dilutions of the pooled cDNA. All the samples were run in duplicates along with the non-template control (NTC). To confirm single gene-specific peaks dissociation protocol with incremental temperatures of 95°C for 15 s plus 65°C for 15 s was used. The qPCR expression data for each gene was extracted in the form of quantification cycle (Cq) and data was subjected for subsequent analysis.

### Evaluation of reference genes (RGs)

For the evaluation RGs expression stability, three different statistical algorithms; geNorm NormFinder [22] and BestKeeper [23] were utilized. Relative Cq values based on comparative Cq-method were the input data for geNorm and Normfinder [2, 24]. The geNorm software defines the gene stability value (M value) by comparing the pairwise variations among the reference genes. In addition, pair wise variation analysis (V values) was carried out using geNorm to select optimal number of RGs to be used for normalization of expression data across buffalo tissues. The contribution of each gene to the variance of normalization factor ratio was calculated to illustrate the effect of adding or removing a particular gene from the final set of RGs. NormFinder algorithm ranks the reference genes by calculating the intra and inter- group variations of expression of each of the reference genes. The BestKeeper analysis included calculation of crossing point standard deviations [{SD, ±CP} <1] and results were displayed as standard deviation (S.D) and coefficient of variance (C.V). The programme has its assumption that the genes which are stably expressed should be highly correlated to each other.

## Results

In the present study, a total of 60 samples of 12 tissue types from 5 adult buffaloes were included to identify panel of RGs for normalization of qPCR data across buffalo tissues. Good quality RNA as reflected by A_260_/A_280_ ratio was extracted from each tissue sample (2.09±0.031). Presence of single melting peak ensured specific amplification of each RG. The co-efficient of determination (R^2^) and efficiency also accounted for qPCR performance (S1 Table and S1–S5 Figs). The qPCR performance in terms of slope of five points standard curves was in the range of -3.138 to -3.646 and showed sufficiently good amplification efficiencies ranging from 88.063% for *UXT* to 109.26% for *RPL4*.

**Fig 1:**
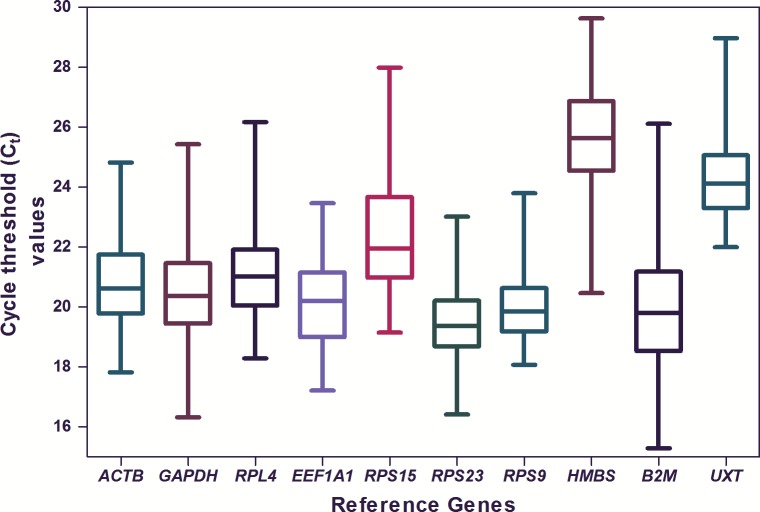
Expression levels of individual candidate RGs. The data is presented as quantification cycle (Cq) values of each gene in the box-whisker diagram. The median is shown as a line across the box while whiskers indicate maximum and minimum values.

The average quantification cycle (Cq) values for 10 RGs were quite variable and ranged from 19.3 for *RPS23* to 25.6 for *HMBS* (Fig 1).

### Expression stability of candidate RGs

Three softwares namely geNorm, NormFinder and BestKeeper were utilized to determine the expression stability of 10 candidate RGs across 12 tissues of riverine buffaloes.

#### Identification of RGs by geNorm analysis

**Fig 2:**
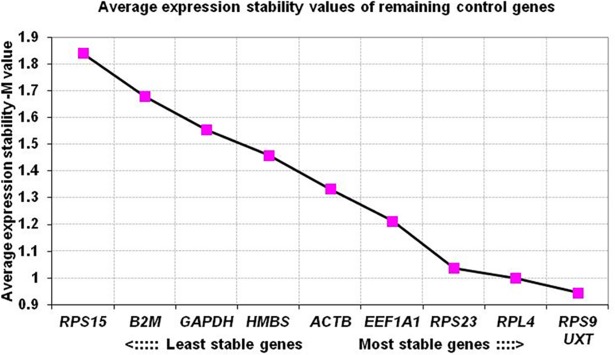
Ranking of RGs based on average expression stability values (M values).

The geNorm software evaluated the expression stability (M value) of each candidate genes and ranked them from the most stable (lowest M value) to least stable (highest M value). On combining the expression data of all 12 tissues, all except *GAPDH*, *β2M* and *RPS15* genes showed their expression stability (M value) within the acceptable range (<1.5). The *M-*value ranged from 0.9707 (*RPS9* and *UXT*) to 1.7362 (*RPS15*) (Table 2). The overall expression stability criteria ranked *RPS9* and *UXT* as most stable genes. The analysis thus classified *GAPDH*, *β2M* and *RPS15* as less suitable RGs for calculation of normalization factor across buffalo tissues (Table 2). Across 12 buffalo tissues, genes were ranked in terms of expression stability index as: *UXT/RPS9*> *RPL4*> *RPS23*> *EEF1A1*> *ACTB*> *HMBS*> *GAPDH*> *β2M*> *RPS15* (Fig 2).

**Table 2:** Ranking of RGs across 12 tissues according to their expression stability by geNorm and NormFinder softwares.

Gene	GeNorm		Normfinder	
	M-value	Rank	Stability value	Rank
*UXT*	0.9707	1	0.475	1
*RPS9*	0.9707	1	0.674	3
*RPL4*	1.0337	2	0.692	4
*RPS23*	1.0646	3	0.621	2
*EEF1A1*	1.1660	4	0.706	5
*ACTB*	1.3098	5	0.838	7
*HMBS*	1.4169	6	0.774	6
*GAPDH*	1.5148	7	0.866	8
*β2M*	1.6099	8	0.934	9
*RPS15*	1.7362	9	1.264	10

**Fig 3:**
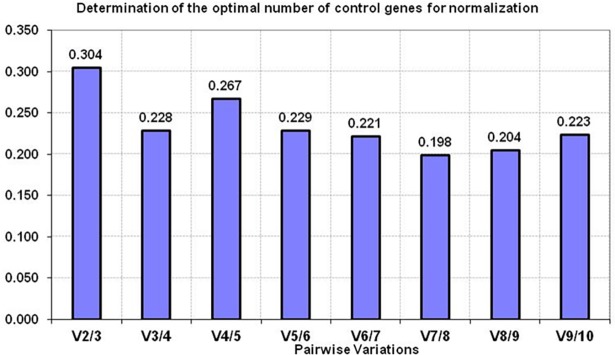
The pairwise variation (V) analysis of candidate RGs for determining the optimal number of genes for normalization across 12 buffalo tissues.

Apart from calculating the expression stability (M) value, the V value was also calculated in different combinations: V2/V3, V3/V4, V4/V5 *etc* by adding the third, fourth and fifth less stable genes. The analysis showed that V2/V3 combination gave slightly higher V value (0.304) than the acceptable limit (recommended cut off value of 0.15) (Fig 3). However, with the addition of *RPS23* in V3/V4 gene combination, some drop in V value was observed (0.228). Though this value was also little higher than the standard acceptable V value, however considering the 12 diverse type of tissues evaluated in the study, the value of 0.228 was considered to be in acceptable range. Few studies [25–27] have also indicated slightly higher V value. In our opinion, for studies handling diverse sample types, V value ≤ 0.15 should be considered as a suggestive threshold criteria and not necessarily a universal cut-off criteria in deciding the number of genes to be used to calculate normalization factor. The V value threshold vary according to the biological system and type of samples being evaluated. Therefore, we used the lowest Vn/n+1 value (0.228) to determine the number of RGs adequate for normalization. Based on the gene stability and pair wise analysis, *UXT*, *RPS9*, *RPL4* and *RPS23* were found to be the appropriate RGs for normalization of expression data across 12 buffalo tissues.

**Fig 4:**
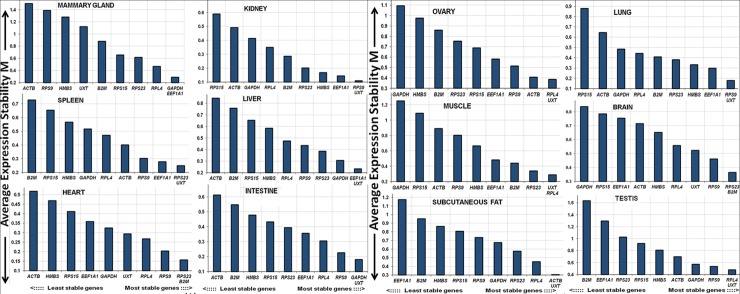
Tissue wise expression stability value (M value) of candidate RGs by geNorm analysis.

In addition to overall analysis, an attempt was made to evaluate the expression stability of candidate RGs in each of the 12 tissue types. The tissue wise geNorm analysis is shown in Fig 4. In majority of the tissues (9 out of 12), *UXT* was found to be most stable and ranked number one (Fig 4 and S2 Table). Similarly *RPL4* and *RPS9*, the two most stable genes identified across 12 tissues; were also ranked higher in several tissue types, indicating their suitability as RGs both within majority tissue types as well as across all tissue types.

**Fig 5:**
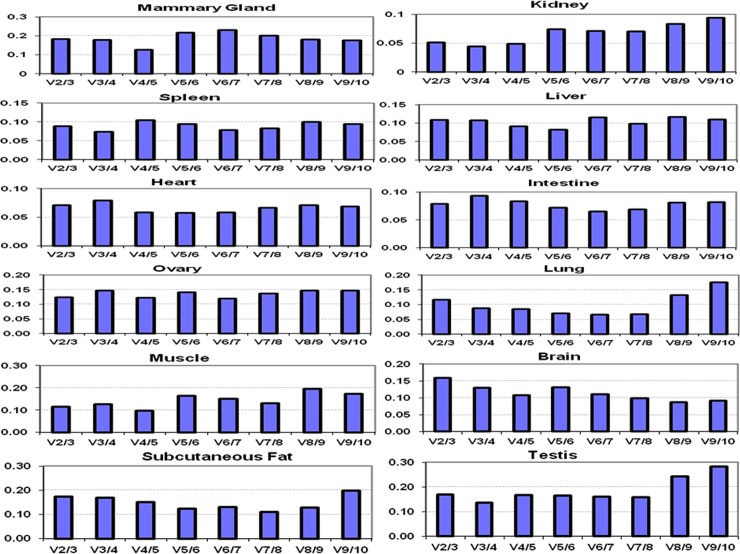
Pairwise variation (V) analysis of 10 RGs to determine optimal number of reference genes for normalization of qPCR data in individual buffalo tissue.

The pair wise analysis was carried out for each tissue to calculate V values. The graphs showing pair wise analysis for individual tissue type is presented in Fig 5. The V2/V3 combination provided V value close to threshold value of 0.15 for each individual tissue. The V values were: mammary gland (0.18), kidney (0.051), spleen (0.089), liver (0.108), heart (0.071), intestine (0.079), ovary (0.123), lung (0.116), muscle (0.114), brain (0.159), subcutaneous fat (0.173) and testis (0.169). Thus, for individual tissue, V2/3 combination gave V value close to the acceptable limit.

#### Selection of RGs by NormFinder analysis

**Fig 6:**
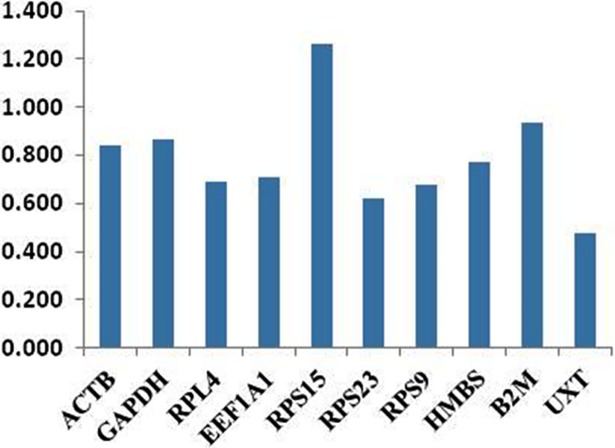
Bar plot showing gene variability across 10 RGs by NormFinder.

For NormFinder analysis, C_q_ values of 10 genes were converted into relative quantities. The NormFinder based gene stability values are shown in Table 2. The Normfinder output was considerably similar to that of geNorm, as same set of genes (*UXT*, *RPS23*, *RPL4*, *RPS9*, *EEF1A1*) was most stable in Normfinder analysis across 12 tissues. Genes like *β2M*, *GAPDH* and *RPS15* showed higher expression variability and were least stable. *UXT* as in geNorm analysis was most stable gene followed by *RPS23*, *RPS9* and *RPL4*. Ranking of genes across 12 tissues from most stable to least stable was as follows: *UXT*> *RPS23*> *RPS9*> *RPL4*> *EEF1A1*> *HMBS*> *ACTB*> *β2M*> *GAPDH*> *RPS15* (Fig 6 and Table 2). Similar to geNorm, gene expression stability was also evaluated using NormFinder tool for individual tissue type. The analysis showed almost similar ranking of RGs in individual tissue as determined in the geNorm analysis (data not shown).

**Fig 7:**
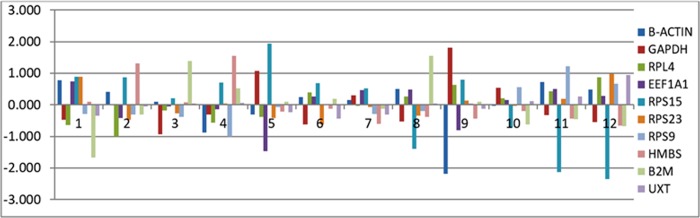
Inter-group variation analysis of RGs across different tissues.

**Fig 8:**
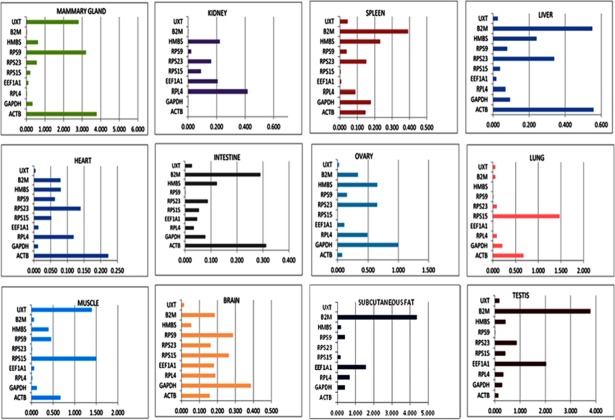
Intra-group variation analysis of candidate RGs in each tissue.

NormFinder also accounted for inter and intra-group variation, as well as the estimation of variances. The inter- and intra- group variation analysis revealed *RPL4*, *RPS23*, *RPS9* and *UXT* genes to be least variable across tissues (Figs 7 and 8). The inter-group variation analysis covering all 12 tissues (mammary gland, kidney, spleen, liver, heart, intestine, ovary, lung, muscle, brain, subcutaneous fat and testis) showed these 4 genes to be more stably expressed genes (Fig 7). On the other hand, in intra-group variation analysis, three most variable genes; *RPS15*, *ACTB* and *B2M* were identified (Fig 8). The *RPS15* expression was variable in lung, brain and muscle; *ACTB* expression was variable in liver, intestine, heart and mammary gland; and *β2M* expression was variable in spleen, liver, intestine, subcutaneous fat and testis. Therefore, these three genes were not classified as suitable RGs in any of the analysis.

#### Evaluation of expression variation by BestKeeper analysis

The gene expression variation based on Cq values for 10 candidate RGs was calculated using BestKeeper algorithm. The *RPS9* gene with lowest crossing point SD value of 0.75 revealed minimum variation (Table 3). This was followed by *UXT*, *RPS23/RPL4*, *GAPDH*, *ACTB*, *EEF1A1* and *HMBS* genes with SD value of 0.95, 0.97, 1.11, 1.13, 1.22 and 1.34 respectively. On the other hand, *β2M* and *RPS15* genes were found to be least stable as variation was greater with SD value of 1.63 and 1.89 respectively. Based on the crossing point SD value and range of fold change expression, BestKeeper analysis has classified genes from most to least stable genes as follows: *RPS9*> *UXT*> *RPS23/RPL4*> *GAPDH*> *ACTB*> *EEF1A1*> *HMBS*> *β2M*> *RPS15*.

**Table 3:** Parameters based quantitative cycle (Cq) values for 10 candidate RGs.

	*ACTB*	*GAPDH*	*RPL4*	*EEF1A1*	*RPS15*	*RPS23*	*RPS9*	*HMBS*	*B2M*	*UXT*
N	54	54	54	54	54	54	54	54	54	54
geo Mean [Cq]	20.68	20.30	20.81	19.94	22.69	19.11	19.74	25.44	19.69	23.93
ar Mean [Cq]	20.72	20.35	20.85	19.99	22.80	19.15	19.76	25.50	19.81	23.96
min [Cq]	17.82	16.32	18.28	17.21	19.15	16.41	18.07	20.47	15.29	21.99
max [Cq]	24.82	22.83	23.67	23.46	27.99	21.72	23.80	28.20	26.11	27.97
Std dev [±Cq]	1.13	1.11	0.97	1.22	1.89	0.97	0.75	1.34	1.63	0.95
CV [%Cq]	5.44	5.46	4.64	6.08	8.30	5.08	3.78	5.25	8.25	3.95
min [x-fold]	-7.25	-15.71	-5.78	-6.63	-11.64	-6.48	-3.18	-31.34	-21.09	-3.83
max [x-fold]	17.68	5.79	7.23	11.49	39.42	6.12	16.62	6.79	86.01	16.49
Std dev [± x-fold]	2.19	2.16	1.96	2.32	3.71	1.96	1.68	2.53	3.11	1.93

N = number of samples, geo Mean[Cq] = geometric mean of Cq; ar Mean[Cq] = arithmetic mean of Cq; min [Cq] and max [Cq] = extreme values of Cq; Std dev [±Cq] = standard deviation of the Cq; CV [%Cq] = coefficient of variation expressed as a percentage on the Cq values; min [x-fold] and max [x-fold] = extreme values of expression levels expressed as absolute x-fold over or under coefficient; std dev[±x-fold] = standard deviation of the absolute regulation coefficients.

Additionally, the inter-gene relationship for 10 RGs pairs was also estimated. Strong correlation coefficients (r) were observed for *UXT/RPS23* (r = 0.654), *RPS23/RPL4* (r = 0.650), UXT/RPS9 (r = 0.642), *RPS9/RPL4* (r = 0.623), *UXT/β2M* (r = 0.575) and *UXT/HMBS* (r = 0.536) (Table 4). This analysis indicated that these pairs of gene have similar expression pattern across various tissues in buffaloes.

**Table 4:** Repeated pair-wise correlation amongst genes with BestKeeper index.

*Pearson correlation coefficient (r)*										
vs.	*ACTB*	*GAPDH*	*RPL4*	*EEF1A1*	*RPS15*	*RPS23*	*RPS9*	*HMBS*	*B2M*	*UXT*
*GAPDH*	-0.359	-	-	-	-	-	-	-	-	-
p-value	0.008	-	-	-	-	-	-	-	-	-
*RPL4*	0.127	0.266	-	-	-	-	-	-	-	-
p-value	0.362	0.052	-	-	-	-	-	-	-	-
*EEF1A1*	*0*.*566*	-0.087	0.404	-	-	-	-	-	-	-
p-value	0.001	0.531	0.002	-	-	-	-	-	-	-
*RPS15*	-0.100	0.525	-0.013	0.078	-	-	-	-	-	-
p-value	0.475	0.000	0.929	0.578	-	-	-	-	-	-
*RPS23*	0.230	0.184	0.650	0.419	0.115	-	-	-	-	-
p-value	0.095	0.183	0.001	0.002	0.410	-	-	-	-	-
*RPS9*	0.237	0.095	0.623	0.274	-0.284	0.415	-	-	-	-
p-value	0.084	0.493	0.001	0.045	0.037	0.002	-	-	-	-
*HMBS*	0.302	0.338	0.189	0.416	0.568	0.390	0.117	-	-	-
p-value	0.027	0.012	0.170	0.002	0.001	0.004	0.399	-	-	-
*B2M*	0.237	0.293	0.423	0.488	0.417	0.448	0.269	0.488	-	-
p-value	0.084	0.032	0.001	0.001	0.002	0.001	0.050	0.001	-	-
*UXT*	0.272	0.226	0.590	0.424	0.053	0.654	0.642	0.536	0.575	-
p-value	0.046	0.101	0.001	0.001	0.705	0.001	0.001	0.001	0.001	-
Best Keeper vs.	*ACTB*	*GAPDH*	*RPL4*	*EEF1A1*	*RPS15*	*RPS23*	*RPS9*	*HMBS*	*B2M*	*UXT*
coeff. of corr. [r]	0.383	0.444	0.639	0.639	0.492	0.703	0.455	0.736	0.808	0.759
p-value	0.004	0.001	0.001	0.001	0.001	0.001	0.001	0.001	0.001	0.001

However, certain pairs of genes showed poor correlation like *RPS9* with *RPS15; ACTB* with *GAPDH*, *RPS15*, *RPL4*; *RPS15* with *EEF1A1*, *RPL4* and *GAPDH* with *EEF1A1*. Further BestKeeper index was calculated for each gene and the correlation between each candidate RG and BestKeeper was estimated. The relationship between RG and BestKeeper was described in terms of Pearson correlation coefficient (r), coefficient of determination (r^2^) and the p value. p<0.05 was obtained for all genes indicating a significant contributions of all genes towards the index. The best correlation between BestKeeper and RG was observed for *β2M* (r = 0.808), *UXT* (r = 0.759) and *HMBS* (r = 0.736) followed by *RPS23* (r = 0.703), *RPL4/EEF1A1* (r = 0.639), *RPS15* (0.492) and *RPS9* (r = 0.481). The high correlation values for these genes indicated their reliability as reference genes except *β2M*, *HMBS* and *EEF1A1* which although showed high correlation value but they showed high x fold change values of 3.11, 2.53 and 2.32 (Table 3) respectively making them unsuitable as RGs. *UXT* was termed as best reference gene based on the highest correlation value and *RPS9* was best RG on the lowest SD and fold change values. The statistically significant correlation shown by these RGs with the BestKeeper index appeared to be consistent with their evaluation as assessed by geNorm and NormFinder.

**Fig 9:**
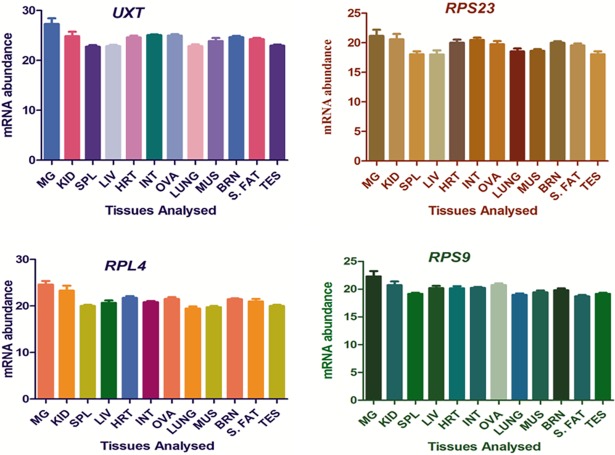
Expression stability of 4 selected RGs (*RPL4*, *RPS23*, *RPS9*, *UXT*) across buffalo tissues. MG-Mammary Gland, KID-Kidney, SPL-Spleen, LIV-Liver, HRT-Heart, INT-Intestine, OVA-Ovary, LUNG-Lung, MUS-Muscle, BRN-Brain, S. FAT-Subcutaneous fat, TES-Testis.

Hence, *UXT*, *RPS23*, *RPL4* and *RPS9* were found to be the most stable RGs with all three algorithms, geNorm, NormFinder and BestKeeper. The expression stability of 4 selected RGs across different tissues was also supported by their near constant Cq values as shown in Fig 9. Thus, in the present study, expression stability of 10 candidate genes from different functional classes were evaluated to select appropriate RGs for qPCR based expression studies in tissues samples of riverine buffaloes.

## Discussion

Numerous studies have shown the importance of RGs to normalize expression data of target genes under specific conditions. Even the choice of one wrong reference gene could affect the overall results [28–29] especially when the used RG is regulated by the experimental conditions. Hence use of multiple RGs has now been considered as appropriate method for accurate normalization [24]. To choose the appropriate genes, number of programs are available like geNorm, BestKeeper and NormFinder that allow the accurate identification of multiple reference genes.

All the three aforesaid strategies were also utilized successfully in the present study to identify the panel of genes with stable expression across different buffalo tissues. Our data showed that *UXT*, *RPS23*, *RPL4* and *RPS9* were the most reliable and stable RGs as identified using all the three algorithms. Further, it was found that *β2M* and *RPS15* were the least stable genes as they showed highest expression variability making them non-suitable RGs for normalization of data. In our study, the ranking of most stable RGs utilizing these different algorithms were comparable to a large extent. In recent past, our groups has conducted number of similar studies to identify appropriate reference gene panels for application in transcriptional studies in Indian native cattle and riverine buffaloes. In one such study, *β2M*, *RPS9* and *RPS15a* genes were identified as best RGs for heat stressed mononuclear cells of Indian cattle and buffaloes [19]; *β2M*, *RPS23*, *RPL4* and *EEF1A1* as most reliable RGs in heat stressed mammary explants and mammary epithelial cells of buffaloes [6, 7]; *RPL4*, *EEF1A1*, *ACTB* and *GAPDH* genes were found to be most stable genes in milk derived mammary epithelial cells in Sahiwal cows during different lactation stages [30]. Similarly identification of stable reference genes for transcriptional studies in bulls distinctive in meat quality [5] and in buffalo muscle tissue [31] were also reported. Terzi and coworkers [32] have followed a different approach wherein, EST transcripts from publicly available *Bos taurus* database were evaluated across different tissues of water buffaloes. In their study, they could identify ribosomal proteins L4, L5 and Gek protein encoding genes as stably expressed transcripts and suggested them to be used as normalizers to compare gene expression levels across buffalo tissues.

In our study, we observed unstable expression of two of the most commonly used RGs, *ACTB* and *GAPDH* across different tissue. In the past, large number of studies have used *GAPDH* and *ACTB* as single control gene [33] to normalize the qPCR data. However several studies have shown that the expression of these reference genes gets affected by the experimental conditions [17]. Hence one should properly evaluate the commonly used genes in any cell type or tissue of interest for correct interpretation of qPCR results.

In summary, the gene expression results might be more reliable if they are normalized by geometric means of multiple reference genes, as recommended in several other studies [13, 24]. Our present data has demonstrated that 2 or more reference genes should be used to validate expression data across buffalo tissues. The results of the present study have provided panel of references that can be utilized during gene expression studies across as well as individual buffalo tissue. In conclusion, *UXT*, *RPS23*, *RPL4*, and *RPS9* were most stable and appropriate reference genes identified across buffalo tissues and their geometric means would provide accurate normalization factor for expression data in buffalo tissues.

## Supporting information

S1 Fig Standard curves, amplification plots and melting peaks for *EEF1A1* and *RPS15*. (TIF)

S2 Fig Standard curves, amplification plots and melting peaks for *B2M* and *HMBS*. (TIF)

S3 Fig Standard curves, amplification plots and melting peaks for *RPL4* and *RPS23*. (TIF)

S4 Fig Standard curves, amplification plots and melting peaks for *RPS9* and *UXT*. (TIF)

S5 Fig Standard curves, amplification plots and melting peaks for *ACTB* and *GAPDH*. (TIF)

S1 Table Gene symbol, slope, PCR efficiency and regression coefficient for the studied RGs. (DOCX)

S2 Table Tissue wise evaluation of expression stability and ranking of each RGs using geNorm. (DOCX)
