# Supplementary figures and images for: Selection of suitable reference genes for normalization of quantitative RT-PCR (RT-qPCR) expression data across twelve tissues of riverine buffaloes (Bubalus bubalis)
Source: PLoS One. 2018 Mar 6;13(3):e0191558. doi: 10.1371/journal.pone.0191558 (PMC5839537; doi:10.1371/journal.pone.0191558)

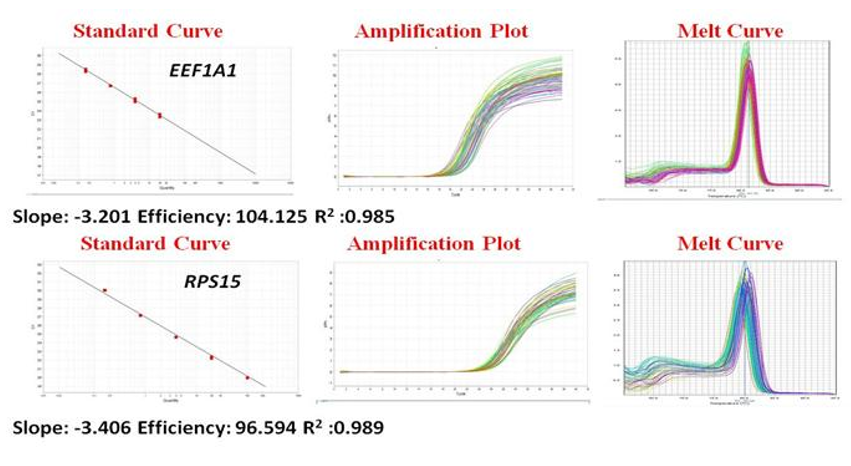

Supplement: S1 Fig — (TIF) [file pone.0191558.s001.tif]

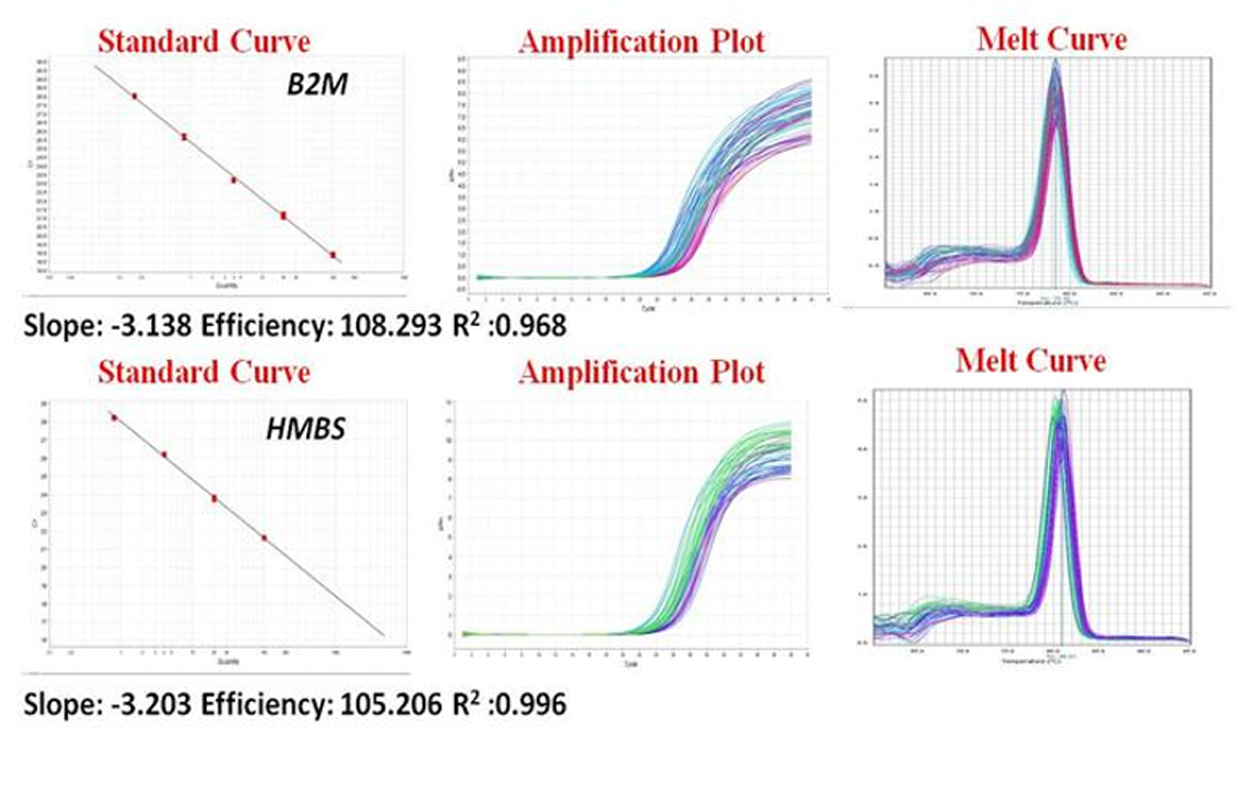

Supplement: S2 Fig — (TIF) [file pone.0191558.s002.tif]

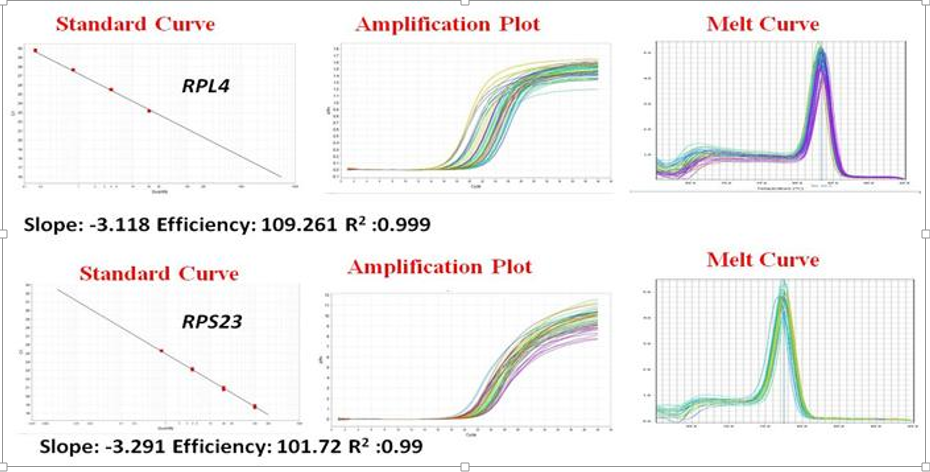

Supplement: S3 Fig — (TIF) [file pone.0191558.s003.tif]

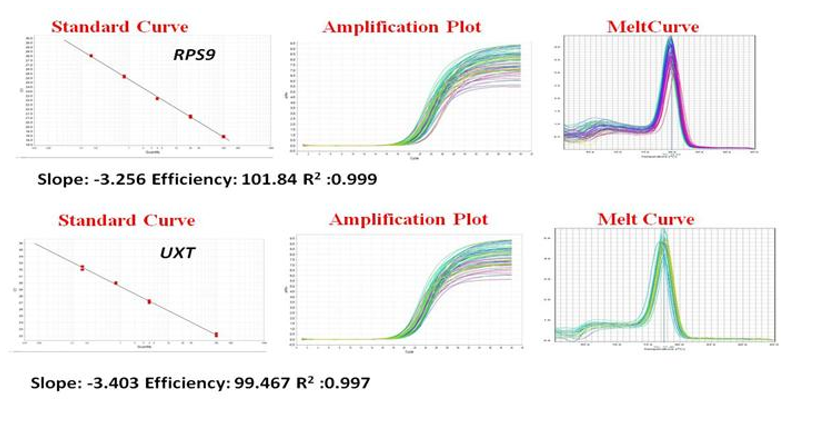

Supplement: S4 Fig — (TIF) [file pone.0191558.s004.tif]

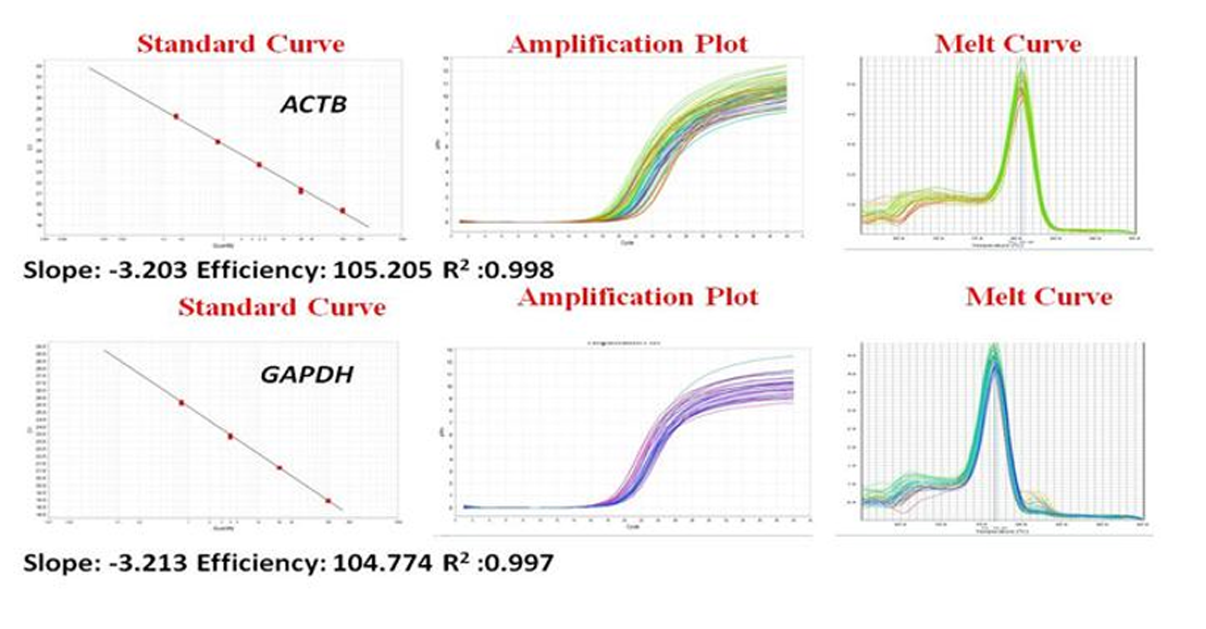

Supplement: S5 Fig — (TIF) [file pone.0191558.s005.tif]
