# Supplementary material for: Selection of suitable reference genes for normalization of quantitative RT-PCR (RT-qPCR) expression data across twelve tissues of riverine buffaloes (Bubalus bubalis)
Source: PLoS One. 2018 Mar 6;13(3):e0191558. doi: 10.1371/journal.pone.0191558 (PMC5839537; doi:10.1371/journal.pone.0191558)
Supplement: S1 Table — (DOCX) [file pone.0191558.s006.docx]

**S1 Table. Gene symbol, slope, PCR efficiency and correlation coefficient for the studied RGs**

| **Gene Symbol** | **Slope** | **PCR Efficiency (%)*** | **R^2^ value**** |
| --- | --- | --- | --- |
| *ACTB* | -3.203 | 105.21 | 0.998 |
| *GAPDH* | -3.213 | 104.77 | 0.997 |
| *EEF1A1* | -3.201 | 104.13 | 0.985 |
| *B2M* | -3.138 | 108.29 | 0.968 |
| *HMBS* | -3.203 | 105.21 | 0.996 |
| *RPL4* | -3.118 | 109.26 | 0.999 |
| *RPS15* | -3.406 | 96.59 | 0.989 |
| *RPS23* | -3.291 | 101.72 | 0.990 |
| *RPS9* | -3.256 | 101.84 | 0.999 |
| *UXT* | -3.403 | 99.47 | 0.997 |

*qPCR efficiencies for each primer pair was calculated from 5 point standard curves using 5 fold dilution series of pooled cDNA from tissue samples **R^2^: correlation coefficient of standard curve slope
