# Supplementary material for: Selection of suitable reference genes for normalization of quantitative RT-PCR (RT-qPCR) expression data across twelve tissues of riverine buffaloes (Bubalus bubalis)
Source: PLoS One. 2018 Mar 6;13(3):e0191558. doi: 10.1371/journal.pone.0191558 (PMC5839537; doi:10.1371/journal.pone.0191558)
Supplement: S2 Table — (DOCX) [file pone.0191558.s007.docx]

**Table S2. Tissue wise evaluation of expression stability and ranking of each RGs using geNorm**

| **Tissue** |  | **geNorm** | | | | | | | | |  |
| --- | --- | --- | --- | --- | --- | --- | --- | --- | --- | --- | --- |
|  |  | ***UXT*** | ***RPS9*** | ***RPL4*** | ***RPS23*** | ***EEF1A1*** | ***ACTB*** | ***HMBS*** | ***GAPDH*** | ***β2M*** | ***RPS15*** |
| Mammary Gland (MG) | M value | 1.12 | 1.38 | 0.46 | 0.61 | 0.29 | 1.5 | 1.28 | 0.29 | 0.87 | 0.65 |
|  | Ranking | 6 | 8 | 2 | 3 | 1 | 9 | 7 | 1 | 5 | 4 |
| Kidney (KID) | M value | 0.11 | 0.11 | 0.35 | 0.20 | 0.14 | 0.50 | 0.17 | 0.41 | 0.29 | 0.60 |
|  | Ranking | 1 | 1 | 6 | 4 | 2 | 8 | 3 | 7 | 5 | 9 |
| Spleen (SPL) | M value | 0.25 | 0.31 | 0.51 | 0.25 | 0.28 | 0.40 | 0.56 | 0.51 | 0.72 | 0.65 |
|  | Ranking | 1 | 3 | 5 | 1 | 2 | 4 | 7 | 6 | 9 | 8 |
| Liver (LIV) | M value | 0.23 | 0.44 | 0.47 | 0.39 | 0.23 | 0.85 | 0.50 | 0.31 | 0.76 | 0.66 |
|  | Ranking | 1 | 4 | 5 | 3 | 1 | 9 | 6 | 2 | 8 | 7 |
| Heart (HRT) | M value | 0.29 | 0.20 | 0.26 | 0.16 | 0.36 | 0.51 | 0.46 | 0.32 | 0.16 | 0.41 |
|  | Ranking | 4 | 2 | 3 | 1 | 6 | 9 | 8 | 5 | 1 | 7 |
| Intestine (INT) | M value | 0.18 | 0.22 | 0.31 | 0.39 | 0.36 | 0.61 | 0.48 | 0.18 | 0.54 | 0.43 |
|  | Ranking | 1 | 2 | 3 | 5 | 4 | 9 | 7 | 1 | 8 | 6 |
| Ovary (OVA) | M value | 0.39 | 0.51 | 0.39 | 0.75 | 0.58 | 0.40 | 0.98 | 1.1 | 0.86 | 0.69 |
|  | Ranking | 1 | 3 | 1 | 6 | 4 | 2 | 8 | 9 | 7 | 5 |
| Lung (LUNG) | M value | 0.18 | 0.18 | 0.44 | 0.38 | 0.30 | 0.64 | 0.33 | 0.48 | 0.40 | 0.87 |
|  | Ranking | 1 | 1 | 6 | 4 | 2 | 8 | 3 | 7 | 5 | 9 |
| Muscle (MUS) | M value | 0.29 | 0.80 | 0.29 | 0.34 | 0.48 | 0.89 | 0.66 | 1.25 | 0.44 | 1.09 |
|  | Ranking | 1 | 6 | 1 | 2 | 4 | 7 | 5 | 9 | 3 | 8 |
| Brain (BRN) | M value | 0.52 | 0.46 | 0.56 | 0.36 | 0.75 | 0.71 | 0.65 | 0.83 | 0.36 | 0.78 |
|  | Ranking | 3 | 2 | 4 | 1 | 7 | 6 | 5 | 9 | 1 | 8 |
| Subcutaneous Fat (S. FAT) | M value | 0.30 | 0.74 | 0.45 | 0.58 | 1.17 | 0.30 | 0.86 | 0.68 | 0.95 | 0.80 |
|  | Ranking | 1 | 5 | 2 | 3 | 9 | 1 | 7 | 4 | 8 | 6 |
| Testis (TES) | M value | 0.48 | 0.54 | 0.48 | 1.02 | 1.30 | 0.69 | 0.80 | 0.57 | 1.63 | 0.92 |
|  | Ranking | 1 | 2 | 1 | 7 | 8 | 4 | 5 | 3 | 9 | 6 |
